# Dendritic Cells Crosspresent Antigens from Live B16 Cells More Efficiently than from Apoptotic Cells and Protect from Melanoma in a Therapeutic Model

**DOI:** 10.1371/journal.pone.0019104

**Published:** 2011-04-28

**Authors:** Diana Matheoud, Camille Baey, Lene Vimeux, Andy Tempez, Michael Valente, Pauline Louche, Agnès Le Bon, Anne Hosmalin, Vincent Feuillet

**Affiliations:** 1 Inserm, U1016, Institut Cochin, Paris, France; 2 Cnrs, UMR8104, Paris, France; 3 Université Paris Descartes, Paris, France; University Paris Sud, France

## Abstract

Dendritic cells (DC) are able to elicit anti-tumoral CD8^+^ T cell responses by cross-presenting exogenous antigens in association with major histocompatibility complex (MHC) class I molecules. Therefore they are crucial actors in cell-based cancer immunotherapy. Although apoptotic cells are usually considered to be the best source of antigens, live cells are also able to provide antigens for cross-presentation by DC. We have recently shown that prophylactic immunotherapy by DC after capture of antigens from live B16 melanoma cells induced strong CD8^+^ T-cell responses and protection against a lethal tumor challenge *in vivo* in C57Bl/6 mice. Here, we showed that DC cross-presenting antigens from live B16 cells can also inhibit melanoma lung dissemination in a therapeutic protocol in mice. DC were first incubated with live tumor cells for antigen uptake and processing, then purified and irradiated for safety prior to injection. This treatment induced stronger tumor-specific CD8^+^ T-cell responses than treatment by DC cross-presenting antigens from apoptotic cells. Apoptotic B16 cells induced more IL-10 secretion by DC than live B16 cells. They underwent strong native antigen degradation and led to the expression of fewer MHC class I/epitope complexes on the surface of DC than live cells. Therefore, the possibility to use live cells as sources of tumor antigens must be taken into account to improve the efficiency of cancer immunotherapy.

## Introduction

Dendritic cells (DC) are professional antigen-presenting cells that are the most powerful stimulators of naive T cells, playing a key role in the initiation of immune responses. They have developed unique cross-presentation pathways allowing major histocompatibility complex (MHC) class I–restricted presentation of antigens of exogenous origin, taken up by endocytosis or phagocytosis. Cross-presentation is crucial for the stimulation of CD8^+^ T lymphocytes and therefore induction of immunity and tolerance to antigens that are not directly synthesized in the cytosol of DC, such as antigens from tumors [1], [2], [3], [4], [5], [6].

Given their unique properties, DC have been used with tumor-associated antigens for active cancer immunotherapy [7]. To date, different sources of antigens have been used to load DC: tumor-specific proteins or synthetic peptides [8], [9], [10], peptides eluted from tumor cell surface HLA molecules [11], apoptotic tumor cells [12], [13], [14], [15], tumor cell lysates [16], [17], [18], tumor cell/DC fusions [19].

Loading MHC class I and MHC class II molecules on DC with peptides derived from defined tumor antigens is the most commonly used strategy for DC vaccination, but this approach presents several disadvantages: the restriction to a limited number of HLA molecules, the limited number of well-defined tumor antigens, the relatively rapid turnover of exogenous peptide-MHC complexes and the induction of a restricted repertoire of T-cell clones. An alternative is to load derivatives from whole tumor cells, as this theoretically allows presentation of all possible epitopes, even if undefined, by all HLA types. The parallel presentation of both class I and II MHC-restricted antigens can promote a stronger overall anti-tumor response and long-term cytotoxic CD8^+^ T-cell memory via CD4^+^ T-cell help [20]. In addition, the requirement for processing results in prolonged antigen presentation [21].

Studies have focused on the use of dead (either apoptotic or necrotic) tumor cells as a source of tumor antigens. In murine models, immunization with DC that had phagocytosed apoptotic/necrotic tumor cells induced good protection against tumors [22], [23]. In patients with different types of cancers, DC loaded with autologous or allogeneic tumor-cells have also been used for immunotherapy [12], [13], [14], [15]. However, despite the elicitation of immune responses, clinical results have not yet matched the hopes raised by preclinical studies. The tumor environment is strongly immunosuppressive by different mechanisms, and only some types of death induce immunogenic stress signals [24], [25], [26].

Besides dead cells, live cells can also be a source of antigen for cross-presentation by DC [27], [28], [29], after antigen uptake through nibbling, a mechanism related to trogocytosis [30]. Recently, we have demonstrated that DC that had acquired antigens from live B16 melanoma cells protected mice from lethal tumor challenge in a prophylactic setting [29]. Here, we assessed the potential of DC loaded with antigens from live tumor cells to control established tumors, and we investigated activation and antigen availability using live tumor cells compared to dead tumor cells.

## Materials and Methods

### Ethics statement

All experiments were performed according to the European Community Council Directive of 11/24th/1986 (86/609/EEC) and with permission of the French Veterinary Services (permit number 75-1321) and approval of the Cochin General Animal Facility Service (accreditation number A-75-14-02). All efforts were made to minimize suffering.

### Mice

Eight to twelve week-old C57Bl/6 mice obtained from Harlan Laboratories were bred in the Cochin Institute specific pathogen-free animal facility.

### Cell culture and purification

B3Z cells and B16 F10 (B16) cells were maintained in complete medium (RPMI 1640 Glutamax supplemented with 10% fetal calf serum, 100 U/ml penicillin, 100 µg/ml streptomycin, 0.1 mM non essential amino acids solution, 10 mM HEPES buffer solution and 1 mM sodium pyruvate, all from Invitrogen). L cells transfected with a form of ovalbumin (OVA) only expressed in the cytoplasm, i.e. OVA-EGFP-DAP (L-OVA cells) were a kind gift from K. Rock (University of Massachusetts Medical School, Boston, MA)[31]. They were cultured in complete medium and 500 µg/ml G418 (Sigma-Aldrich). B3Z cells, *lacZ*-inducible CD8^+^ T cell hybridoma which express β-galactosidase upon specific recognition of the OVA_257-264_ (SIINFEKL)-H-2K^b^ complex [32], were a kind gift from C. Leclerc (Institut Pasteur, France).

To avoid caspase-mediated cell death, cells were cultured in the presence of 10 µM z-VAD FMK (Sigma-Aldrich). Alternatively, apoptosis was induced by γ-irradiation (100 Gy), then cells were used after 48-hour incubation in complete medium. The proportion of annexin V^+^PI^-^ apoptotic cells was ≥70%.

For bone marrow-derived DC (BMDC) generation, bone marrow cells from C57Bl/6 mice were depleted of red blood cells using 0.84% ammonium chloride. Cells (2×10^5^/ml) were resuspended in complete medium with 30 µl/ml of hybridoma supernatant containing granulocyte-macrophage colony-stimulating factor (kind gift from David Gray, Royal Postgraduate Hospital, London). Medium was replaced after 2 and 4 days. Immature DC were obtained after 6 days.

For in vitro cross-presentation assays, BMDC were cultured for 16 h with different amounts of live or apoptotic cells in the presence of lipopolysaccharide (LPS from *E.Coli* 0111:B4; 500 ng/ml; Sigma-Aldrich) and DC were purified using CD11c microbeads for positive immunomagnetic selection (Miltenyi Biotec). Purified DC were cultured for 18 h with the B3Z-T cell hybridoma. After addition of the CPRG substrate, optical density was measured at 560 nm by an ELISA reader (Berthold CB911).

DC maturation was assessed after incubation with live or apoptotic B16 cells with or without LPS and IFNγ (20 ng/ml, Roche). Twenty-four hours later, supernatants were collected to assess cytokine production by ELISA and DC were used for flow cytometry.

### Anti-tumor protection *in vivo*

On day 0, mice were injected i.v. with 10^6^ B16 cells to induce lung tumors. DC were cultured for 16 hours in the presence of LPS (500 ng/ml) and IFNγ (20 ng/ml), either alone or with B16 derived MHC class I-restricted synthetic peptides (gp100_25–33_ and TRP2_181–188_, 10 µM each), or with live B16 cells in the presence of z-VAD, or with apoptotic B16 cells. DC (5×10^5^) were then washed, purified, γ-irradiated (100 Gy) to eliminate any residual live tumor cells, and immediately injected i.v into C57Bl/6 mice on days 3 and 10. On day 15, tumor nodules were counted on the lung surface. Splenocytes were restimulated for 36 hours with γ-irradiated B16 cells or 1 µM peptide (gp100_25–33_ or TRP2_181–188_). The ability of stimulated splenocytes to produce IFNγ was assayed by ELISPOT or flow cytometry.

### Flow cytometric analysis

DC maturation was evaluated by immunofluorescence labeling with the following mAb: anti-CD11c-PE-Cy7, anti-CD80-FITC, anti-CD86-APC, anti-IA^b^-PE. T cells were labeled with the following mAb: anti-CD3-PE-Cy7, anti-CD4-biotin, anti-CD8α-APC-H7 and anti-IFNγ-APC. The anti-CD11c-PE-Cy7 mAb was from eBiosciences, the others from BD. Labeling was performed at 4°C using PBS containing 5% fetal calf serum and 5 mM EDTA (Sigma). Cells were incubated with purified anti-CD16/32 (2.4G2) mAb to block Fc receptors (BD) for 20 min. Then they were labeled with conjugated antibodies for further 20 min. For biotinylated antibodies, streptavidin-PerCP (BD Biosciences) was added in a second step for 15 min. For intracellular IFNγ labeling, spleen cells were restimulated for 4 hours at 37°C in 5% CO2 at 10^6^ cells/ml in complete medium with 0.5 µg/ml phorbol myristate acetate (PMA) and 0.5 µg/ml ionomycin in the presence of Golgi Plug (BD), and labeled with anti-CD3, anti-CD4, anti-CD8 mAb, then intracellularly with an anti-IFNγ mAb (Intracellular staining kit, BD). Annexin-V staining was performed using the Annexin-V apoptosis detection kit from BD Bioscience. Events were acquired using a FACS CANTO or a BD LSR II flow cytometer and analyzed using Diva (Version 6.1.1, BD Biosciences) followed by FlowJo (Version 7.2.5; TreeStar).

### ELISPOT assays

PVDF microplates (Millipore) were coated overnight at 4°C with 4 µg/ml of an anti-mouse IFNγ rat monoclonal antibody (AN18, Mabtech AB) diluted in PBS. After extensive washes, wells were blocked with RPMI containing 10% AB human serum (ABCYS) for 2 hours at 37°C. Three ×10^5^ splenocytes were incubated overnight with one of the peptides corresponding to a specific epitope (final concentration 10 µg/ml) or with B16 melanoma cells. The plates were then washed, incubated with 1 µg/ml biotinylated anti-mouse IFNγ rat monoclonal antibody (R4-6A2, Mabtech AB) diluted in PBS for 2 hours at 37°C, and then overnight at 4°C. Plates were subsequently incubated with extravidine-coupled alkaline phosphatase (Sigma-Aldrich) diluted in PBS. After adding the BCIP/NBT substrate (Sigma-Aldrich), IFNγ spot forming cells were counted using a BioReader 5000F ELISPOT reader and expressed as the number of spots per million cells tested.

### ELISA

Culture supernatants were assayed for IL-12p70 and IL-10 using kits from Ebioscience according to supplier instructions. Optical density was measured at 560 nm using an ELISA Berthold CB911 reader.

### Western Blots

Live or apoptotic B16 and L cells were scraped at 4°C in CHAPS buffer (1% 3-[(3-Cholamidopropyl)dimethylammonio]-1propanesulfonate, 10 mM Hepes pH 7.4, 150 mM NaCl, 10% glycerol; Sigma-Aldrich) containing proteases inhibitors (Complete EDTA-free tablets, Roche) and 0.2 mM sodium orthovanadate (Sigma-Aldrich). Protein concentration was measured by spectrometry at 280 nm (NanoDrop, Labtech). Cell lysates were boiled at 95°C for 5 min in sample application buffer containing 2-mercaptoethanol (Lane Marker Reducing Sample buffer; Thermosciences). Fifty µg of proteins (for B16 cells), or 25 µg of proteins (for L cells), from each sample were separated by SDS/10%PAGE (ready-to-use; BioRad) for 1 hour at 150 V and transferred onto a nitrocellulose membrane for 1 hour at 80 V. Membranes were then blocked for 2 hours with TBS/0,05% Tween buffer plus 5% milk (BioRad). Membranes were probed with a polyclonal rabbit antibody to OVA (for OVA-EGFP-DAP cells), a polyclonal goat antibody to gp100, a polyclonal goat antibody to TRP2 (for B16 cells) or a polyclonal goat antibody to actin overnight at 4°C and incubated with horseradish peroxidase-conjugated antibodies to rabbit IgG or to goat IgG (all antibodies from Santa Cruz Biotechnology) for 1 hour. Visualization was carried out with enhanced chemiluminescence (GE Healthcare). Bands were analyzed using a LAS 300S reader.

### Statistical analysis

The significance of differences between series of results was assessed by unpaired *t* test (Prism 5, GraphPad Software). P values<0.05 were considered significant.

## Results

### DC loaded with live melanoma cells induced protection against B16 tumors in a therapeutic setting and a stronger CD8^+^ T cell response than DC cultured with apoptotic melanoma cells

**Figure 1:**
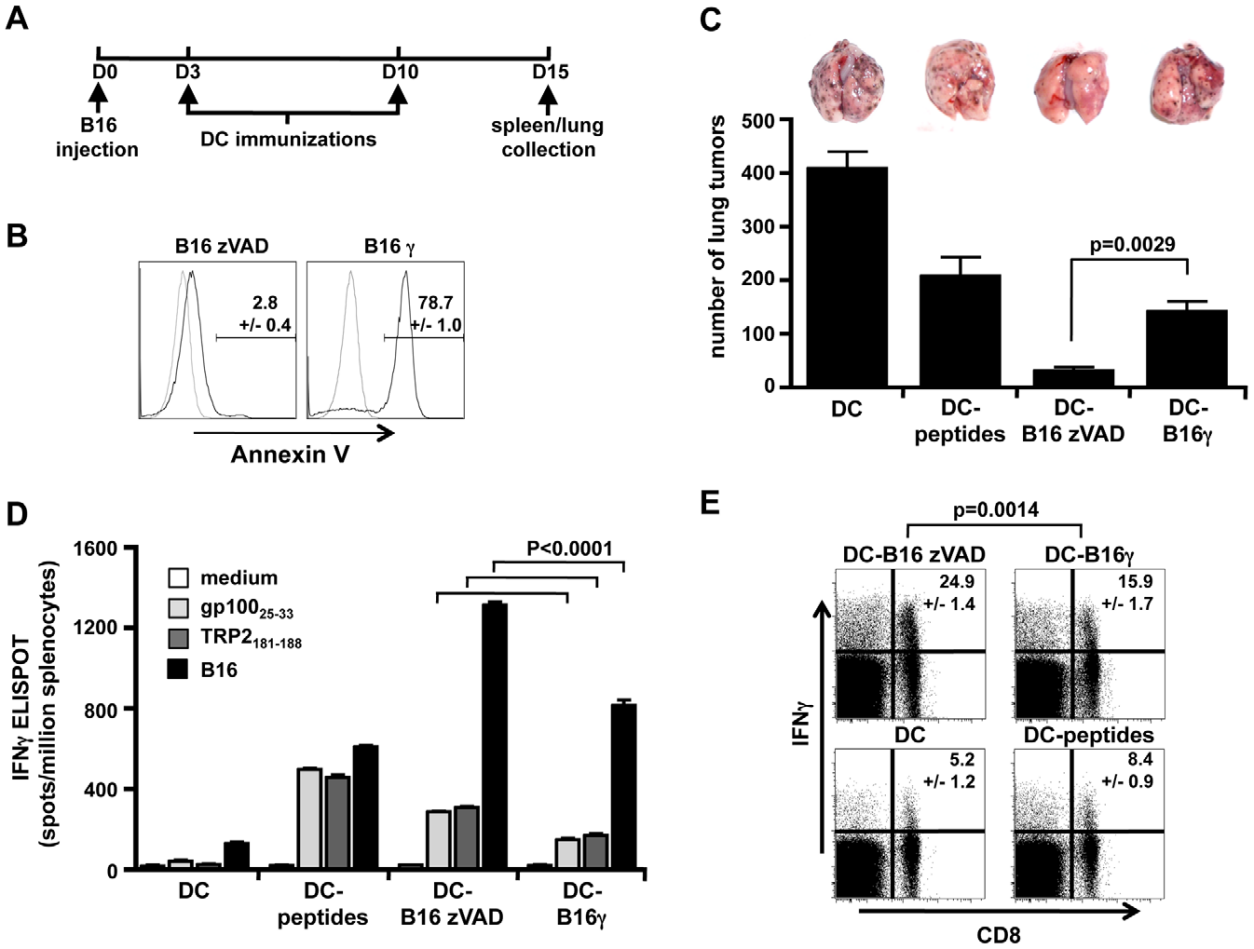
DC cultured with live B16 cells induced strong protection against tumor in a therapeutic setting. A, time schedule outlining the different stages as used in the experiments. Five C57BL/6 mice per group were inoculated *i.v.* with 1.10^6^ B16 cells at day 0. At days 3 and 10, mice were immunized with 0,5.10^6^ of purified and irradiated DC that were previously cultured for 16 h in the presence of LPS and IFNγ, with either culture medium (DC), or gp100_25–33_ and TRP2_181–188_ peptides (DC-peptides), or live (zVAD treated) B16 cells (DC-B16 zVAD) or apoptotic (γ-irradiated) B16 cells (DC-B16γ). B, B16 cell apoptosis was evaluated by annexin-V staining (solid lines) compared to no staining (dotted lines) and the mean number ± SEM of the Annexin-positive events is shown. At day 15, mice were sacrificed, lung tumors were counted (C) and T cell responses were evaluated in the spleen. D, Splenocytes were restimulated with gp100_25–33_ or TRP2_181–188_ peptides or B16 cells or culture medium and tested in an IFNγ ELISPOT assay. E, After B16 restimulation, splenocytes were also stimulated with PMA and ionomycin and then labeled with anti-CD3, anti-CD8 and intracellularly with anti-IFNγ antibodies. Events were gated on CD3^+^CD8^+^ splenocytes and IFNγ production was mesured by flow cytometry. The mean ± SEM of data from 5 mice per group are shown for an experiment representative of two independent experiments. The significance of differences between series was assessed by unpaired *t* test.

After reporting that DC cultured with live B16 melanoma cells induced a strong protection against a lethal tumor challenge in a preventive immunization model [29], we tested this immunization in a therapeutic setting (Fig. 1A). At day 0, C57BL/6 mice were inoculated *i.v.* with B16 cells. Bone marrow-derived DC were cultured for 16 h in the presence of LPS and IFNγ, with either live B16 cells and the caspase inhibitor zVAD to avoid caspase-mediated apoptosis (DC-B16 zVAD), or apoptotic B16 cells (DC-B16γ) (Fig. 1B), or tumor specific MHC class I-restricted peptides (gp100_25–33_ and TRP2_181–188_, DC-peptides), or medium alone (DC). DC were purified to 98% by positive selection, irradiated at 100 Gy to avoid injecting any residual live tumor cell, then injected *i.v.* 3 and 10 days after tumor inoculation. At day 15, mice treated with DC alone had 406±50 tumors in their lungs, whereas mice treated with DC cultured with live B16 cells were strongly protected as they only displayed 28±14 tumors. Surprisingly, protection was more efficient using DC cultured with live rather than with apoptotic tumor cells (Fig. 1C, p = 0.0029). IFNγ responses to gp100_25–33_ or TRP2_181–188_ or to B16 tumor cells were reproducibly higher with DC-B16 zVAD than with DC-B16γ (Fig. 1D). As expected, immunization with DC cultured with B16 cells induced higher responses to the tumor itself than immunization with DC-peptides. Immunization with peptide-loaded DC yielded similar numbers of IFNγ positive spots in response to either B16 cells or to peptide-loaded DC, whereas immunization with live or apoptotic cell-loaded DC yielded much higher responses to B16 cells than to peptide-loaded DC, indicating stimulation of a larger set of clones responding to a broader antigenic repertoire, including responses to TRP2_181–188_ and gp100_25–33_ (Fig. 1D). Indeed, B16 cells provide many more tumor epitopes, including undefined epitopes, than the two peptides. IFNγ responses to B16 cells were induced in CD8^+^CD3^+^ T cells in the spleens from mice immunized with DC-B16 specifically compared to spleens from mice immunized with DC or DC-peptides (Fig. 1E), showing MHC-class I-restricted responses related to cross-presentation. Responses were also induced in CD8^−^CD3^+^, CD4^+^ T cells (not shown). Therefore, DC cultured with live tumor cells, purified and strongly irradiated to prevent inoculation of proliferating tumor cells, induced more efficient protection and a stronger immunity in a therapeutic protocol than DC cultured with apoptotic tumor cells.

### Culture of DC with apoptotic B16 cells led to higher IL-10 production than culture with live B16 cells

**Figure 2:**
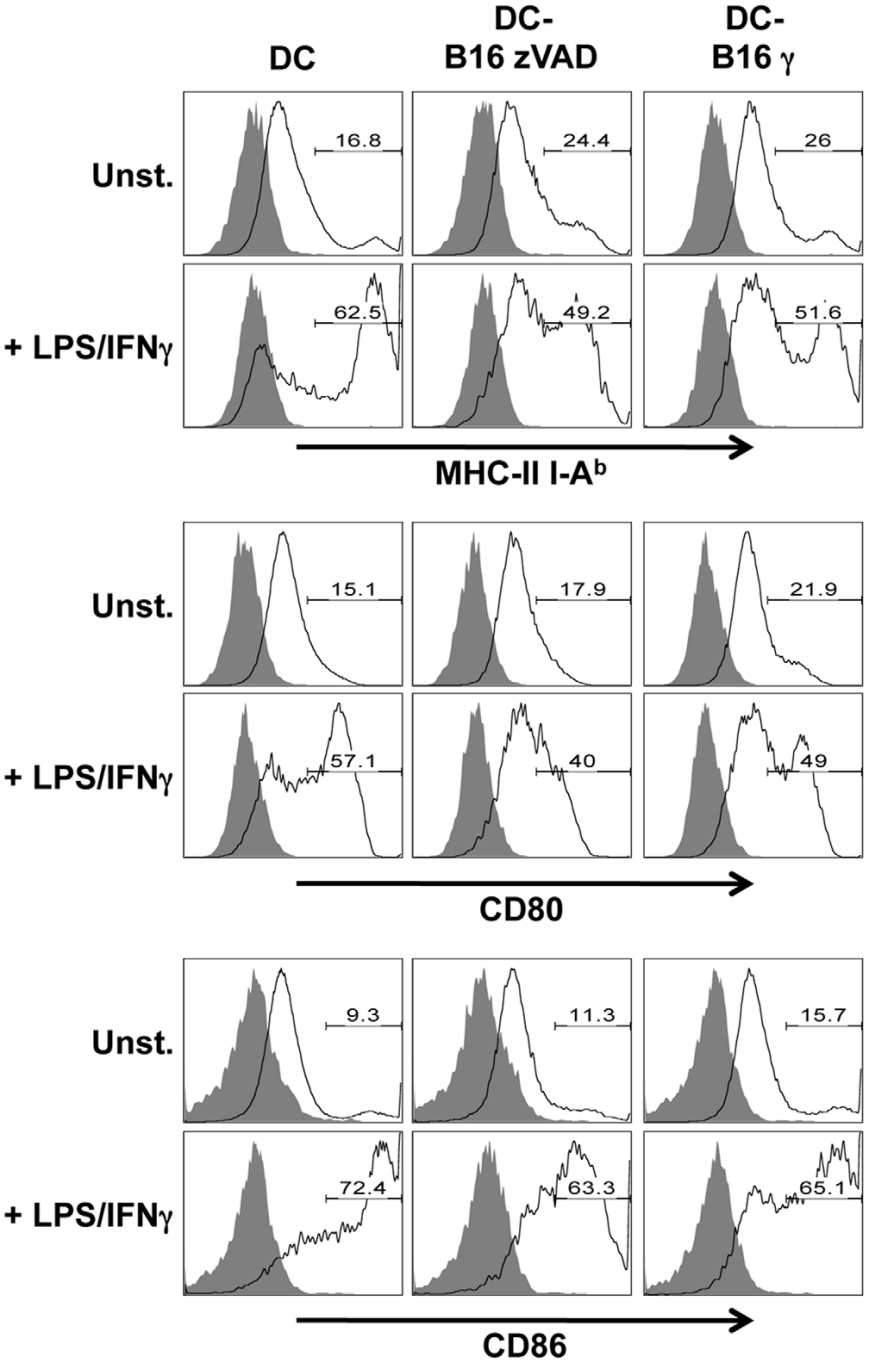
DC maturation after culture with live or apoptotic B16 cells. DC were cultured with live (zVAD treated) or apoptotic (γ-irradiated) B16 cells for 24 h in the presence of culture medium or LPS and IFNγ. The expression of maturation molecules was then tested by flow cytometry. One representative experiment out of three independent experiments is shown.

**Figure 3:**
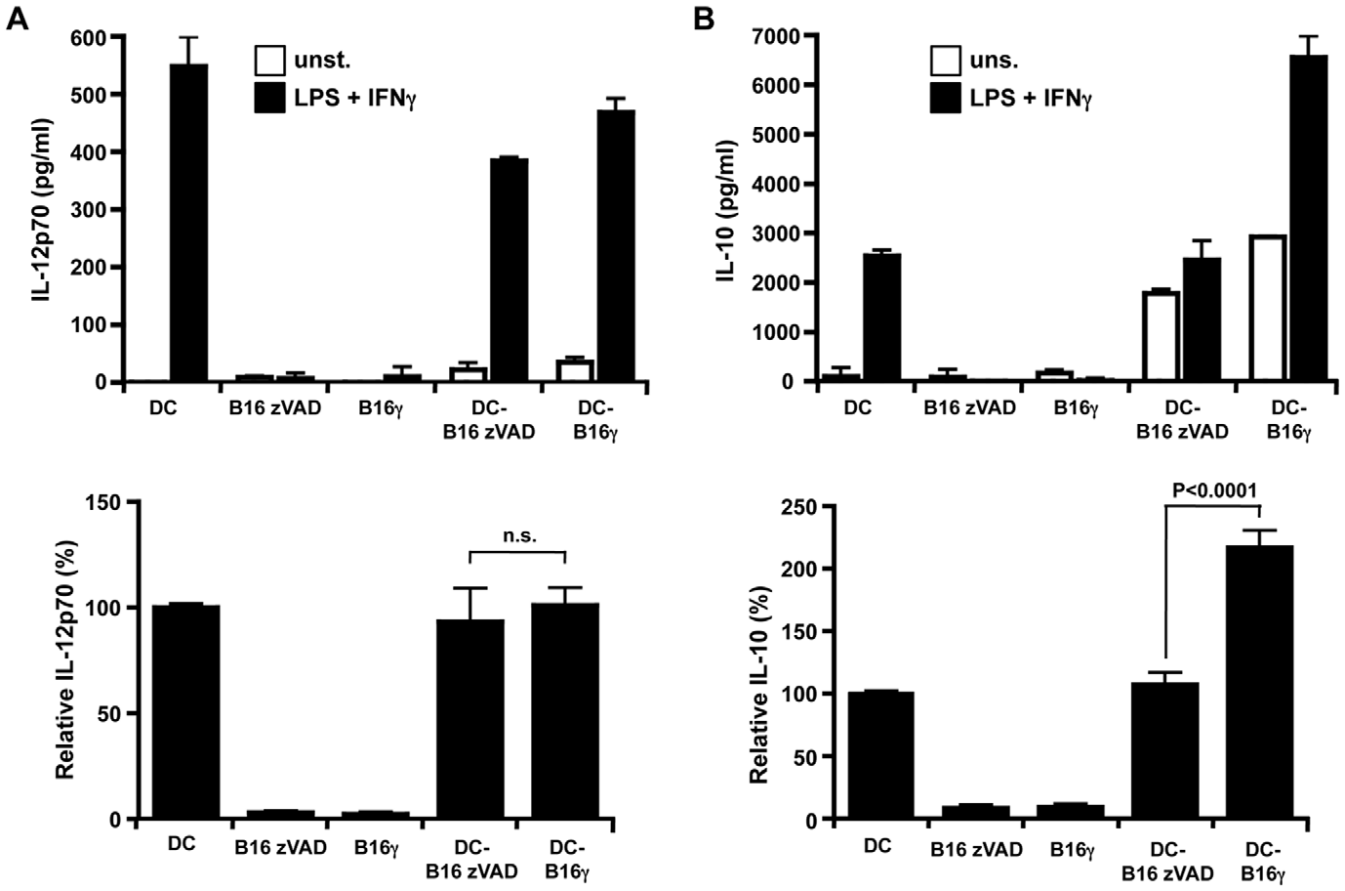
DC cytokine profile after culture with live or apoptotic B16 cells. DC were cultured alone (DC) or with live (zVAD treated) or apoptotic (γ-irradiated) B16 cells for 24 h and stimulated or not with LPS and IFNγ. As controls, B16 zVAD and B16γ cells were also cultured alone. DC were then assessed for their ability to produce IL-12p70 (A) or IL-10 (B) by ELISA. In the upper panels, data from one out of three independent experiments are shown. In the bottom panels, for LPS and IFNγ stimulation, relative IL-12p70 and IL-10 productions are expressed as a percentage of the cytokine production obtained for DC alone. Mean percentage values±SEM are shown. The significance of differences between series of results was assessed using a paired t test (n = 3, 3 independent experiments).

We next documented the impact of culture with either live or apoptotic B16 cells on DC maturation. DC were cultured with live (zVAD treated) or apoptotic (γ-irradiated) B16 cells for 24 h while maturation was induced or not using LPS and IFNγ. First, DC maturation was assessed by labeling for MHC class II molecules and the costimulatory CD80 or CD86 molecules. Without stimulation, DC cultured either with live cells or apoptotic cells displayed a phenotype consistent with a slight maturation. On the contrary after stimulation with LPS and IFNγ, DC cultured with apoptotic or live B16 cells were less mature than DC cultured alone. The maturation phenotype of DC was slighly higher after culture with apoptotic than with live B16 cells (Fig. 2). In the same cultures, we also examined the production of the proinflammatory cytokine IL-12 (p70) and the anti-inflammatory cytokine IL-10. As expected, live or irradiated B16 cells did not produce any IL-12p70 or IL-10 even after stimulation with LPS and IFNγ. DC cultured with live or apoptotic cells produced similar concentrations of IL-12p70 after stimulation with LPS and IFNγ (Fig. 3A). On the contrary, DC cultured with apoptotic B16 cells produced more IL-10 than DC cultured with live B16 cells or cultured alone (Fig. 3B). Therefore, culture of DC with apoptotic B16 cells compared to live B16 cells led to slightly more phenotypic maturation, and significantly more IL-10 secretion, which might decrease CD8^+^ T lymphocyte stimulation.

### Live cells improved cross-presentation by DC by keeping antigens in a more native form than apoptotic cells

**Figure 4:**
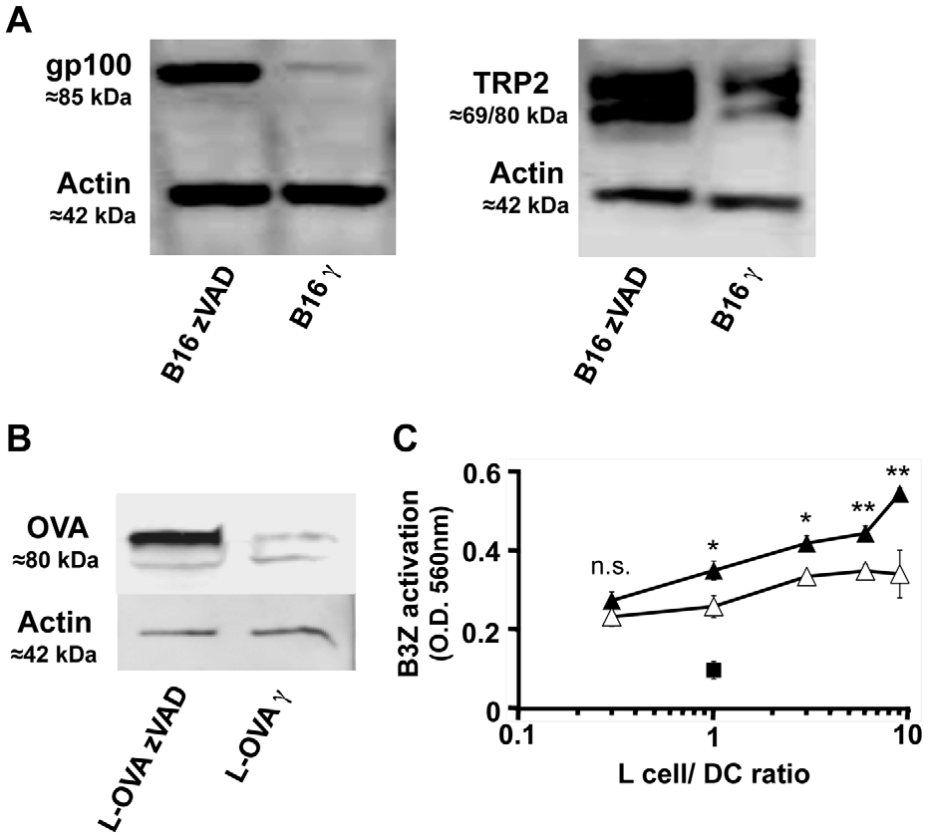
More native antigen in live than in apoptotic donor cells: improved antigen crosspresentation by DC. A, B, 30.10^6^ live (zVAD treated) or apoptotic (γ-irradiated) B16 cells (A) or L-OVA cells (B) were lysed for protein extraction. Lysates from B16 cells were analysed using anti-gp100, anti-TRP2 and anti-actin antibodies (A). Lysates from L-OVA cells were analysed using anti-OVA and anti-actin antibodies antibodies (B). Anti-actin antibodies were used as controls. C, DC were cultured with different numbers of live (filled triangle) or apoptotic (open triangle) L-OVA cells or live L cells (filled square). DC were then purified by magnetic sorting using anti-CD11c microbeads and cultured with B3Z-T cells hybridoma cells for 18 h. Activation after recognition of the SIINFEKL-K^b^ complex was detected by optical density measurement at 560 nm after addition of the CPRG substrate. The significance of differences between series of results was assessed using a two-tailed unpaired t test. **p<0.01, *p<0,05, n.s. not significant. Mean ± SEM, representative of two independent experiments.

One parameter that controls cross-presentation efficiency is the amount of antigen accessible to the DC [33]. We performed western blots to evaluate the quality and the amount of two tumor antigens, gp100 and TRP2, in live or apoptotic B16 cells. We observed that both antigens were strongly degraded in apoptotic melanoma cells, while they were conserved in a native form in live cells (Fig. 4A). To examine the impact of the conservation of native antigen on cross-presentation, we used L cells expressing cytosolic, non-secreted ovalbumin (L-OVA) as a source of antigen, so as to evaluate the expression level of MHC class I-CD8^+^ T cell OVA epitope (SIINFEKL) complexes on DC with the B3Z hybridoma. We could not use OVA-expressing B16 cells since they expressed a secreted form of OVA. We first confirmed that, similarly to gp100 and TRP2 in B16 cells, OVA antigen was strongly degraded in apoptotic L-OVA cells, while it was conserved in a native form in live cells (Fig. 4B). After culture of DC with live or apoptotic L-OVA cells at different ratios, cross-presentation was then evaluated using the B3Z hybridoma. About three times more apoptotic cells than live cells were necessary to reach the maximal level of MHC class I-SIINFEKL complexes, which was lower using apoptotic cells. Thus, B3Z activation was significantly higher when DC were cultured with live than with apoptotic antigen donor cells. Therefore, better native antigen conservation in live cells compared to apoptotic cells seems to induce the expression of a higher number of MHC class I-epitope complexes on DC.

## Discussion

We have previously reported that cross-presentation of antigens from live B16 melanoma cells by DC induced a strong protection against a lethal tumor challenge in a preventive immunization model [29]. Here, we showed that injection of DC loaded with live B16 melanoma cells also protected mice from tumor dissemination in a therapeutic model. In the prophylactic immunization model, T cell responses were consistently stronger when DC were cultured with live melanoma cells than with apoptotic cells [29]. Here in the therapeutic model, DC cultured with live tumor cells induced more efficient protection and a stronger immunity than DC cultured with apoptotic tumor cells. We investigated what might confer a stronger immunogenicity to live cells.

The ability of DC to trigger an effective T cell response is dependent on their state of maturation and on cytokine production. We observed slightly higher MHC and costimulatory molecule expression levels after culture of DC with apoptotic compared to live cells, but similar IL-12 production levels. IL-12 is required for the polarization of Th1 responses, which in turn promote the activation of CD8^+^ T cells into effector cytotoxic T lymphocytes, allowing the clearance of infected or transformed cells. On the contrary, IL-10, mainly secreted by DC and regulatory T cells, is rather a tolerogenic cytokine, which can inhibit T cell activation. [34]. DC cultured with apoptotic cells produced more IL-10 than DC cultured with live cells, conferring them potential tolerogenic properties, particularly when DC were matured with LPS and IFNγ, which corresponds to the maturation stimulus used in our immunotherapeutic protocol.

The conservation state of antigens is a very important parameter in DC-based immunotherapy since the induction of an effective T cell response depends on the quality and quantity of the antigen [33]. It has already been shown that antigens are degraded in apoptotic bodies [35]. Moreover, it has also been proposed that pre-processing of antigens in apoptotic or autophagic cells could improve their cross-presentation by DC [36], [37]. Here, western blots showed that antigens were strongly degraded in apoptotic cells, while they were conserved in a native form in live cells. The degradation of antigens in apoptotic cells could have an impact on cross-presentation, making it less effective. Indeed, a dose-response experiment showed that the number of MHC class I-SIINFEKL complexes on DC was greater with live cells than with apoptotic cells as antigen-donor cells. Thus, the impact of the conservation status of antigen on cross-presentation may be considered in two ways. On the one hand, death by apoptosis or autophagy could provide pre-processed antigens, almost “ready-to-use” for DC [38], [39]. On the other hand, our data show that live cells can be a source of native antigen, the correct processing of which would be under full control of the specialized DC.

Until recently, only apoptotic cells were considered as an effective cellular source of antigen for cross-presentation, and immunization protocols using DC loaded with whole tumor cells have been carried out with apoptotic tumor cells, neglecting the possibility to use live tumor cells. In a preliminary prophylactic immunization experiment where we injected purified, but unirradiated DC before tumor challenge, we suspected that despite protection against the challenge, a larger tumor in an immunized mouse than in controls might be related to a contaminating tumor cell in the DC preparation. Consequently, we chose to use strongly irradiated DC to prevent inoculation of proliferating tumor cells, and ensure safety. Irradiation of the DC did not preclude antitumor protection nor T cell responses. Therefore, live tumor cells should be taken into account as a source of antigens to load DC and improve the efficiency of tumor immunotherapy protocols, because they supply native tumor antigens that can be efficiently processed by specialized DC for cross-presentation.
